# Under the Weather with Ciguatera Fish Poisoning: Climate Variables Associated with Increases in Suspected Cases

**DOI:** 10.1289/ehp.122-A167

**Published:** 2014-06-01

**Authors:** Julia R. Barrett

**Affiliations:** Julia R. Barrett, MS, ELS, a Madison, WI–based science writer and editor, has written for *EHP* since 1996. She is a member of the National Association of Science Writers and the Board of Editors in the Life Sciences.

Ciguatera fish poisoning (CFP), the most common nonbacterial illness linked with eating fish, arises from the ciguatoxin produced by *Gambierdiscus* algae in tropical and subtropical areas of the Pacific, Western Atlantic, and Indian oceans.^1^ In this issue of *EHP* investigators assess the relationship between climate variability and reports of CFP, concluding that incidence of CFP is likely to increase with rises in sea-surface temperature and tropical storm frequency.^1^

Ciguatoxin bioaccumulates up the food chain as herbivorous fish eat the algae and are themselves eaten by carnivorous fish.^2^ The odorless, tasteless toxin withstands freezing and cooking, and people who ingest it begin experiencing gastrointestinal, neurological, and sometimes cardiovascular symptoms within hours of exposure. The symptoms usually resolve after a few days but may linger for months or years, or return after apparent recovery; death is rare. An estimated 50,000–500,000 cases of CFP occur annually worldwide.^3^ CFP is thought to be a widely unreported illness.^4^

**Figure:**
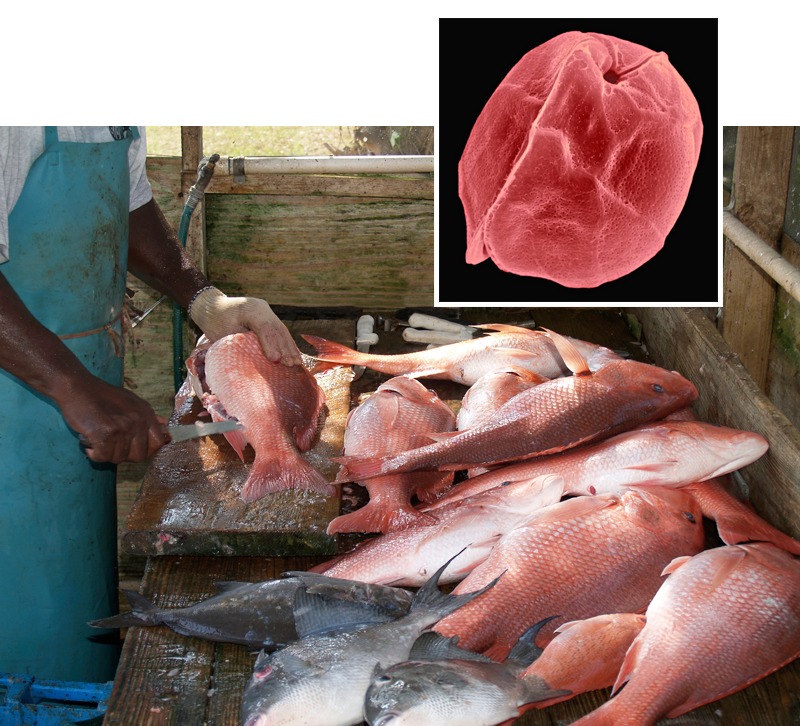
Gambierdiscus algae (inset) produce a toxin that contaminates reef fish such as red snapper, triggerfish, and mackerel. Fish: © LindaJohnsonbaugh/iStockphoto; Gambierdiscus: © Dennis Kunkel Microscopy, Inc./Visuals Unlimited/Corbis

Climate change is predicted to increase sea-surface temperatures in some areas, which could allow harmful marine algae to become more prolific over a wider territory.^4^^,^^5^^,^^6^ In the current study, the researchers hypothesized that CFP incidence increases in conjunction with warmer sea-surface temperatures and increased tropical storm activity.

“What we were interested in is the extension of ciguatera into the mainland United States, either from fish importing or through expansion of the endemic area up the sea coast,” says Daniel Gingold, who led the study at Emory University. “It’s possible that the biggest change [in incidence] that would happen due to climate change is not actually in places where it’s already endemic—it may occur in places that used to not be able to support ciguatera but now do.”

Information on possible CFP cases during 2001–2011 came from the National Poison Data System; the data comprised 1,102 calls to poison control centers in the continental United States, Puerto Rico, and the U.S. Virgin Islands. Cases were grouped by month and analyzed with monthly sea-surface temperatures and severe tropical storm activity in the Caribbean during 1999–2011. The model incorporated lag times of 0, 3, 6, 12, 18, and 24 months between weather events and reported CFP cases.

Lyndon Llewellyn, research manager at the Australian Institute of Marine Science, characterized these lag times as an important aspect. “There will often be a delay between the initiation of ciguatera flowing through the ecosystem before human poisonings are observed,” he says. Llewellyn was not involved in the study.

Based on their analysis, the investigators estimated that each additional storm in a month was associated with an 11% increase in CFP-related calls 18 months later, and that each 1°C increase in maximum sea-surface temperature during the month of August was associated with a 62% increase in calls after a lag of 5–16 months.^1^

These results were then considered in light of predictions about climate change. Assuming a 2.5°C increase in maximum Caribbean sea-surface temperature and a 10% increase in storm frequency, the researchers estimated an additional 239 CFP-related calls per year. The maximum estimated increase was 461 calls.^1^

There are numerous caveats to the findings. “The sea-surface temperature projections in the tropical Atlantic by our climate models are really bad,” says Vasu Misra, an associate professor of meteorology at the Center for Ocean-Atmospheric Prediction Studies, The Florida State University, who was not involved with the study. “So, attribution of climate to CFP incidence is a very difficult task.” Misra also notes a growing consensus that overall Atlantic storm frequency may go down in the future climate, although more intense storms may become slightly more frequent.

Additionally, it was impossible to confirm CFP cases, fish origin, and location of exposure, and factors such as tourism and changes in fish consumption over time were not controlled.^1^ “We are missing a lot of information about this whole question,” says Gingold. “It’s been hypothesized for a long time that ciguatera would be affected by climate. But we really don’t have enough data to answer this question yet—we don’t have enough years of data, and we don’t have a very good surveillance system for identifying the toxin in commercial fish or for confirming actual cases.”

Whether as an emerging disease in nonendemic areas or a neglected tropical disease in endemic areas, ciguatera and *Gambierdiscus* algae both warrant scrutiny.^2^ The limitations in this study generally pervade ciguatera research, says Llewellyn, who adds, “This paper is a good example of what is needed—biomedical professionals, especially epidemiologists, focusing on ciguatera.”
